# A phase 1b study of humanized KS-interleukin-2 (huKS-IL2) immunocytokine with cyclophosphamide in patients with EpCAM-positive advanced solid tumors

**DOI:** 10.1186/1471-2407-13-20

**Published:** 2013-01-15

**Authors:** Joseph P Connor, Mihaela C Cristea, Nancy L Lewis, Lionel D Lewis, Philip B Komarnitsky, Maria R Mattiacci, Mildred Felder, Sarah Stewart, Josephine Harter, Jean Henslee-Downey, Daniel Kramer, Roland Neugebauer, Roger Stupp

**Affiliations:** 1 University of Wisconsin, Madison, WI, 53706, USA; 2 City of Hope, Duarte, CA, USA; 3 Kimmel Cancer Center of Thomas Jefferson University, Philadelphia, PA, USA; 4 Department of Medicine and The Norris Cotton Cancer Center, Dartmouth Medical School and Dartmouth–Hitchcock Medical Center, Lebanon, NH, USA; 5 EMD Serono Inc, Rockland, MA, USA; 6 Merck Serono S.A. – Geneva, Geneva, Switzerland; 7 Merck KGaA, Darmstadt, Germany; 8 University of Lausanne Hospitals (CHUV), Lausanne, Switzerland; 9 Presently at Actelion Pharmaceutical Ltd, Allschwil, Switzerland

**Keywords:** huKS-IL2, Immunocytokine, Solid tumors

## Abstract

**Background:**

Humanized KS-interleukin-2 (huKS-IL2), an immunocytokine with specificity for epithelial cell adhesion molecule (EpCAM), has demonstrated favorable tolerability and immunologic activity as a single agent.

**Methods:**

Phase 1b study in patients with EpCAM-positive advanced solid tumors to determine the maximum tolerated dose (MTD) and safety profile of huKS-IL2 in combination with low-dose cyclophosphamide. Treatment consisted of cyclophosphamide (300 mg/m^2^ on day 1), and escalating doses of huKS-IL2 (0.5–4.0 mg/m^2^ IV continuous infusion over 4 hours) on days 2, 3, and 4 of each 21-day cycle. Safety, pharmacokinetic profile, immunogenicity, anti-tumor and biologic activity were evaluated.

**Results:**

Twenty-seven patients were treated for up to 6 cycles; 26 were evaluable for response. The MTD of huKS-IL2 in combination with 300 mg/m^2^ cyclophosphamide was 3.0 mg/m^2^. At higher doses, myelosuppression was dose-limiting. Transient lymphopenia was the most common grade 3/4 adverse event (AE). Other significant AEs included hypotension, hypophosphatemia, and increase in serum creatinine. All patients recovered from these AEs. The huKS-IL2 exposure was dose-dependent, but not dose-proportional, accumulation was negligible, and elimination half-life and systemic clearance were independent of dose and time. Most patients had a transient immune response to huKS-IL2. Immunologic activity was observed at all doses. Ten patients (38%) had stable disease as best response, lasting for ≥ 4 cycles in 3 patients.

**Conclusion:**

The combination of huKS-IL2 with low-dose cyclophosphamide was well tolerated. Although no objective responses were observed, the combination showed evidence of immunologic activity and 3 patients showed stable disease for ≥ 4 cycles.

**Trial registration:**

http://NCT00132522

## Background

Cell adhesion molecules, such as epithelial cell adhesion molecule (EpCAM; CD326), play a pivotal role in the pathogenesis of cancer [1]. EpCAM is expressed on the surface of epithelial cells where it is involved in the onset, development, maintenance, repair, and homeostatic functions of epithelia. As a homotypic cell adhesion molecule, EpCAM is closely integrated within the WNT and cadherin–catenin pathways. It has recently been shown to modulate the expression of proto-oncogenes, such as c-myc [2].

Initially described as a tumor-associated antigen, EpCAM is highly expressed in gastrointestinal, lung, prostate, breast, ovarian, and other cancers of epithelial origin [3-7]. Its overexpression in these tumors may be considered as a factor that disrupts the regulatory balance and facilitates aberrant cellular proliferation, differentiation, migration, and intracellular signaling processes that underlie tumor growth and metastasis [1]. Interestingly, the prognostic impact of EpCAM expression varies with tumor type [8,9]. The pleiotropic activity of EpCAM, together with high surface expression in some tumor types, makes it a natural candidate for targeting with therapeutic antibodies or antibody–drug conjugates.

A variety of approaches have been investigated to therapeutically target EpCAM-expressing tumors, including anti-EpCAM scFv–*Pseudomonas* exotoxin A fusion construct evaluation in patients with squamous cell carcinoma of the head and neck [10], vaccination with EpCAM protein to induce EpCAM-specific T-cell responses in patients with colorectal carcinoma [11], and a number of monoclonal, bi-specific and tri-specific anti-EpCAM antibody therapies [12-16].

Humanized KS-interleukin-2 (huKS-IL2) is an immunocytokine conjugate consisting of a humanized antibody specific for EpCAM linked at its Fc end to 2 molecules of interleukin-2 (IL2). The EpCAM antibody component of huKS-IL2 targets IL2 to EpCAM-positive tumors for the generation of cytotoxic T-cells and activation of the innate immune system, i.e. natural killer (NK) cells, in the tumor microenvironment. In preclinical studies, huKS-IL2 demonstrated significant anti-tumor effects when administered intravenously or directly into EpCAM-positive tumors [17]. Preclinical data provide a rationale for evaluating huKS-IL2 in combination with other therapies, such as radiofrequency ablation or low-dose cyclophosphamide, with both therapies augmenting the anti-tumor response induced by huKS-IL2 [17,18]. The observed synergy with low-dose cyclophosphamide is believed to be due to downregulation of regulatory T-cells, thus enhancing with the immunomodulatory effect of IL2.

When administered as a single agent, huKS-IL2 was well tolerated in a phase 1 study of patients with advanced prostate cancer, which defined a maximum tolerated dose (MTD) of huKS-IL2 of 6.4 mg/m^2^[19]. The present phase 1b study aimed to assess the safety and to determine the MTD of huKS-IL2 administered following a single low-dose of cyclophosphamide in patients with EpCAM-expressing advanced solid cancers. Pharmacokinetic (PK) profile, immunogenicity, anti-tumor and biologic activity were also evaluated.

## Methods

### Study objectives

The primary objectives of this multicenter, open-label, phase 1 study were to assess the safety and tolerability, and determine the MTD of huKS-IL2 administered following a single low dose of cyclophosphamide in patients with EpCAM-positive advanced cancers. Secondary objectives were to characterize the PK profile of huKS-IL2 after cyclophosphamide, to study its effects on immunogenicity and immunologic function, and to observe survival and anti-tumor activity. The study protocol was approved by the local Institutional Review Board (IRB)/Independent Ethics Committee at each participating center and the regulatory authorities, as appropriate (University of Wisconsin Health Sciences IRB, Committee for Protection of Human Subjects of Dartmouth College, the Fox Chase Cancer Center IRB, and City of Hope IRB). The study was conducted in accordance with Good Clinical Practice and the ethical principles of the Declaration of Helsinki. All patients gave their written informed consent prior to study entry.

### Patient selection

Patients (age ≥ 18 years) with advanced or recurrent solid tumors were eligible after failing standard therapy. Tumor tissue had to demonstrate EpCAM expression by immunohistochemistry on ≥ 25% of the tumor cells, as centrally assessed. Other eligibility criteria included: Karnofsky Performance Status score ≥ 70%, adequate baseline organ function, as defined by aspartate transaminase, alanine transaminase ≤ 2.5 × the upper limit of normal (ULN), bilirubin ≤ 1.5 × ULN, no history of significant renal impairment or chronic kidney disease, normal creatinine, or creatinine clearance ≥ 60 mL/min, adequate pulmonary function (≥ 70% of predicted values for forced vital capacity and forced expiratory volume in 1 second, O_2_ saturation ≥ 90%) unless due to malignant disease to be treated, no significant impairment of hematopoietic and cardiac functions, and blood sodium, potassium, and phosphorus within normal limits. The major study exclusion criteria were known brain metastases, immediate need for palliative radiotherapy or systemic corticosteroid therapy, immunosuppression, autoimmune disease (except autoimmune thyroiditis and vitiligo). Pregnancy or lactation, known serious uncontrolled medical condition, known hypersensitivity to study drugs or excipients, or prior exposure to huKS-IL2, were also exclusion criteria. Patients had to be able to safely discontinue concomitant antihypertensive therapy for at least 48 hours, and to tolerate concomitant treatment with indomethacin or other nonsteroidal anti-inflammatory drugs.

### Treatment

Eligible patients received up to 6 cycles of cyclophosphamide (300 mg/m^2^ intravenous [IV] on day 1), followed by 4-hour IV infusions of huKS-IL2 (EMD 273066, EMD Pharmaceuticals, Inc., Durham, NC) on days 2, 3, and 4 at escalating doses (0.5, 1.0, 2.0, 3.0, and 4.0 mg/m^2^), with cycles repeated every 21 days. Study medication huKS-IL2 was supplied as frozen concentrated solution (1 mg/mL) in single-dose glass vials with sufficient overage to remove a 4-mL dose, which was admixed with 25 mL of 0.9% sodium chloride prior to infusion. Patients were sequentially assigned to a specific dose level, no intra-patient dose escalation was allowed. MTD was defined as the dose level, comprising at least 6 patients, immediately below the dose that elicited a dose-limiting toxicity (DLT) in at least 2 out of 3 or 6 patients enrolled at a given dose level. A DLT event was defined as a grade 3 or 4 adverse drug reaction according to the National Cancer Institute Common Terminology Criteria for Adverse Events version 3.0 (NCI CTCAE v 3.0) that occurred during the first cycle. Escalation to the next dose cohort was allowed if a DLT occurred in < 1 of 3 or < 2 of 6 patients treated.

### Outcome measures

#### Safety and tolerability

Adverse events (AEs) and laboratory values were monitored continuously throughout the study and were graded by severity and relationship to study drug. Blood samples for hematology, chemistry, and assessment of immunologic function were collected before or after huKS-IL2 dosing on cycle days 1–5, 9, and 16 for cycles 1 and 2; on cycle days 1, 9, and 16 for cycles 3–6; and within 30 days of treatment discontinuation.

#### Pharmacokinetics of huKS-IL2

Blood samples for huKS-IL2 PK analysis were collected before and after infusion on days 2–5 of each cycle. Serum huKS-IL2 concentrations were measured using a validated enzyme-linked immunosorbent assay (ELISA) method at the bio-analytical laboratory of BioProof AG, Munich, Germany. This assay had a lower limit of quantification of 25 ng/mL in neat serum. It showed an inter-batch precision at the 3 quality control levels between 1.0% and 4.2% and inter-batch accuracy between 90.6% and 93.7%. Serum concentrations were then merged with final clinical data (administration, randomization, demographics, and sampling information) and subjected to noncompartmental PK analysis for PK parameter estimation using the validated software tool Kinetica®, version 4.4.1 at Merck Serono, Department of Exploratory Medicine, Darmstadt, Germany. Key PK parameters determined included area under the concentration–time curve (AUC) from time zero to 24 hours after the start of infusion (AUC_0–24h_), maximum or peak serum concentration (C_max_), AUC from time zero to infinity (AUC_0–∞_), total body clearance (CL), and apparent terminal half-life (t_1/2_).

#### Immunogenicity

To evaluate immunogenicity, blood samples were drawn on days 1 and 2 (before infusion of cyclophosphamide and huKS-IL2, respectively) and days 8 and 16 of cycle 1, and days 1 and 8 of subsequent cycles. A bridging ELISA assay to detect and quasi-quantify antibodies directed against huKS-IL2 in human serum samples was developed and validated. Murine KS (for anti-idiotype antibodies), huFc-IL2 (for anti-Fc-IL2 antibodies) and IL2 cleaved from huKS-IL2 (for anti-IL2 antibodies) were used as capture reagents. In all 3 assays, biotin-conjugated huKS-IL2 was used for detection. The immune response against huKS-IL2 was quasi-quantified by back-calculating the obtained optical densities to a calibration curve consisting of a serial dilution of a polyclonal goat-anti-huKS-IL2 antiserum.

#### Biologic activity, anti-tumor response, and survival

Peripheral blood mononuclear cell (PBMC) populations from whole blood were enumerated using flow cytometric analysis during the first 2 cycles of treatment. *In vitro* T-cell activity in response to tetanus toxoid protein was assessed. Tumor response was evaluated according to Response Evaluation Criteria in Solid Tumors (RECIST, version 1.0) following cycles 2, 4, and 6 and at the time of study completion, if the last course of treatment was not the 2nd, 4th, or 6th cycle. Survival was assessed by patient follow-up for 1 year after the last treated patient’s first dose of huKS-IL2.

### Statistical analysis

Data were analyzed using descriptive statistics. The safety population included all patients who received at least 1 dose of huKS-IL2 or cyclophosphamide and who had at least 1 follow-up visit for safety assessment. The MTD population comprised patients who completed cycle 1 of treatment. Unless discontinuation was due to DLT, patients withdrawn before cycle 1 completion for other reasons were replaced. The efficacy population included all patients who received at least 1 dose each of huKS-IL2 and cyclophosphamide, and who had at least 1 post-baseline assessment of tumor status. The PK population included all patients who had completed at least 1 infusion of huKS-IL2 and who provided all planned PK samples on day 2 of cycle 1. Overall survival was analyzed according to the Kaplan–Meier method from day 1 of therapy until death or last follow-up; this analysis was performed only on treatment arms with at least 6 patients enrolled and on the overall group. Dose proportionality of huKS-IL2 was assessed by comparing AUC_0–24h_ and C_max_ on day 2 of cycle 1 with the various absolute administered doses (mg) using linear regression analysis. Peak and trough concentrations of huKS-IL2 in serum and AUC_0–24h_ were compared within and between cycles for evidence of drug accumulation. Before study initiation, up to 30 patients were planned for enrollment with a sample size of 3–6 patients per dose cohort for each of 5 dose cohorts.

## Results

### Patient demographics

From May 2005 to January 2008, 27 patients (10 men and 17 women) with a median age of 58 (range 46–69) years were enrolled.

Patient baseline demographics are shown in Table 1. The most common tumor histology was ovarian cancer, which was diagnosed in 15 (56%) patients. Other tumor types were lung cancer in 4 (15%) patients, colon cancer in 3 (11%) patients, prostate cancer in 2 (7%) patients, and 1 patient each with melanoma, acinic cell carcinoma, and adenocarcinoma of unknown origin. All but 2 patients had received prior chemotherapy.

**Table 1:** Patient baseline demographics

**Patient characteristic**	**huKS-IL2 dosing cohort, mg/m**^**2**^					**Total (N = 27)**
	**0.5**	**1.0**	**2.0**	**3.0**	**4.0**	
	**(n = 3)**	**(n = 4)**	**(n = 7)**	**(n = 6)**	**(n = 7)**	
Age, years						
Median	47	55	59	57	63	58
Range	47–58	46–69	46–68	49–61	53–67	46–69
Sex, n (%)						
Men	0	1 (25)	4 (57)	1 (17)	4 (57)	10 (37)
Women	3 (100)	3 (75)	3 (43)	5 (83)	3 (43)	17 (63)
Tumor type, n (%)						
Ovarian carcinoma	3 (100)	2 (50)	3 (43)	4 (67)	3 (43)	15 (56)
Lung carcinoma	0	0	0	2 (33)	2 (29)	4 (15)
Colon	0	2 (50)	1 (14)	0	0	3 (11)
Prostate	0	0	1 (14)	0	1 (14)	2 (7)
Other^a^	0	0	2 (29)	0	1 (14)	3 (11)
Tumor stage, n (%)						
I	0	0	1 (14)	0	1 (14)	2 (7)
II	0	1 (25)	0	0	0	1 (4)
III	3 (100)	3 (75)	1 (14)	4 (67)	2 (29)	13 (48)
IV	0	0	5 (71)	2 (33)	4 (57)	11 (41)
Prior chemotherapy, n (%)	3 (100)	4 (100)	6 (86)	6 (86)	6 (86)	25 (93)
Prior radiotherapy, n (%)	0	2 (50)	3 (43)	3 (43)	3 (43)	10 (37)

^a^ Other includes 1 patient each of melanoma, acinic cell carcinoma, and adenocarcinoma of unknown origin.

*huKS-IL2*, humanized KS-interleukin-2.

### Drug exposure

The median time on study was 25 days (range, 1–109 days), with patients exposed to median cumulative doses of 12 mg/m^2^ huKS-IL2 (range, 1–54 mg/m^2^) and 1,182 mg/m^2^ cyclophosphamide (range, 440–3,841 mg/m^2^).

### Safety and adverse events

Treatment-emergent adverse events (TEAEs) were observed in all 27 patients. Common, and mostly mild/moderate, TEAEs were nausea (82%), pyrexia (74%), chills (70%), rash (56%), fatigue (48%), diarrhea (41%), vomiting (41%), and headache (41%). Severe grade 3 and 4 AEs were observed in 14 patients (52%) and considered treatment- and dose-related in 11 patients (Table 2). Five of 7 (71%) patients assigned to the 4.0 mg/m^2^ dosing cohort experienced a treatment-related grade 3 or 4 TEAE compared with 2 of 6 (33%) patients in the 3.0 mg/m^2^ cohort, and 2 of 7 (29%) patients in the 2.0 mg/m^2^ cohort. Most common treatment-related grade 3 or 4 TEAEs were lymphopenia and hypophosphatemia (Table 2).

**Table 2:** Treatment-related NCI CTCAE Version 3.0 Grade 3 or 4 treatment-emergent adverse events

**System organ class, n (%)**	**huKS-IL2 dosing cohort, mg/m**^**2**^					**Total (n = 27)**
	**0.5**	**1.0**	**2.0**	**3.0**	**4.0**	
	**(n = 3)**	**(n = 4)**	**(n = 7)**	**(n = 6)**	**(n = 7)**	
Any treatment-related TEAE of NCI CTCAE Grade 3 or 4	2 (67)	0	2 (29)	2 (33)	5 (71)	11 (41)
Blood/lymphatic system disorders						
Lymphopenia	1 (33)	0	1 (14)	2 (33)	1 (14)	5 (19)
Anemia	0	0	0	0	1 (14)	1 (4)
Neutropenia	0	0	0	0	1 (14)	1 (4)
Thrombocytopenia	0	0	0	0	1 (14)	1 (4)
WBC count decreased	0	0	0	0	1 (14)	1 (4)
Investigations						
Blood phosphorus equivalents increased	0	0	0	0	1(14)	1 (4)
GGT increased	0	0	0	1 (17)	0	1 (4)
Gastrointestinal disorders						
Ascites	1 (33)	0	0	0	0	1 (4)
Nausea	0	0	0	0	1 (14)	1 (4)
Vomiting	0	0	0	0	1 (14)	1 (4)
Metabolism/nutrition disorders						
Hypophosphatemia	0	0	1 (14)	0	1 (14)	2 (7)
Respiratory/thoracic/mediastinal disorders						
Dyspnea	0	0	0	1 (17)	0	1 (4)
Bronchospasm	0	0	0	1 (17)	0	1 (4)
Hypoxia	0	0	1 (14)	0	0	1 (4)

*CTCAE*, Common Terminology Criteria for Adverse Events; *GGT*, gamma-glutamyltransferase; *huKS-IL2*, humanized KS-interleukin-2; *NCI*, National Cancer Institute; *TEAE*, treatment-emergent adverse event; *WBC*, white blood cell.

Other TEAEs classified as being of particular clinical importance included 3 instances of grade 2 transient/reversible hypotension (0.5, 1.0, and 4.0 mg/m^2^ cohorts). Another patient, in the 2.0 mg/m^2^ cohort, experienced a reversible grade 2 increase in serum creatinine lasting 3 days that was considered serious. In all patients, all toxicities improved to a lower grade or resolved.

### Dose-limiting toxicities

Four patients experienced a total of 7 DLTs during cycle 1 (Table 3). These DLTs were hypoxia, dyspnea, bronchospasm, gamma-glutamyltransferase (GGT) increased, thrombocytopenia, neutropenia, and anemia. With the exception of grade 3 dyspnea and GGT increase in a single patient lasting 25 and 19 days, respectively, all other DLTs resolved within 2 or 3 days. The MTD was exceeded following huKS-IL2 dosing at 4.0 mg/m^2^ at which level 2 patients experienced DLTs of thrombocytopenia/neutropenia and anemia. Six patients were dosed with 3.0 mg/m^2^, with 1 patient experiencing DLT events of dyspnea, bronchospasm, and elevated GGT. One out of 7 patients in a 2 mg/m^2^ dose cohort experienced a DLT of severe hypoxia. The MTD of huKS-IL2 in combination with cyclophosphamide was determined to be 3.0 mg/m^2^.

**Table 3:** Patients with dose-limiting toxicity events

**huKS-IL2 dosing cohort, mg/m**^**2**^	**Patient age, years**	**DLT**	**AE grade**^**a**^	**Day started (cycle)**	**Duration, days**	**DLT outcome**
2.0	63	Hypoxia	3	2 (1)	2	Recovered
3.0	59	Dyspnea	3	3 (1)	25	Recovered
		Bronchospasm	3	4 (1)	2	Recovered
		GGT increased	3	4 (1)	19	Recovered
4.0	55	Thrombocytopenia	4	8 (1)	3	Recovered
		Neutropenia	3	8 (1)	3	Recovered
	63	Anemia^b^	3	5 (1)	2	Recovered

^a^ National Cancer Institute Common Terminology Criteria for Adverse Events grade.

^b^ Patient entered study with anemia, but was hospitalized after dosing for a blood transfusion.

*DLT*, dose-limiting toxicity; *GGT*, gamma-glutamyltransferase; *huKS-IL2*, humanized KS-interleukin-2.

### Pharmacokinetics of huKS-IL2

**Figure 1:**
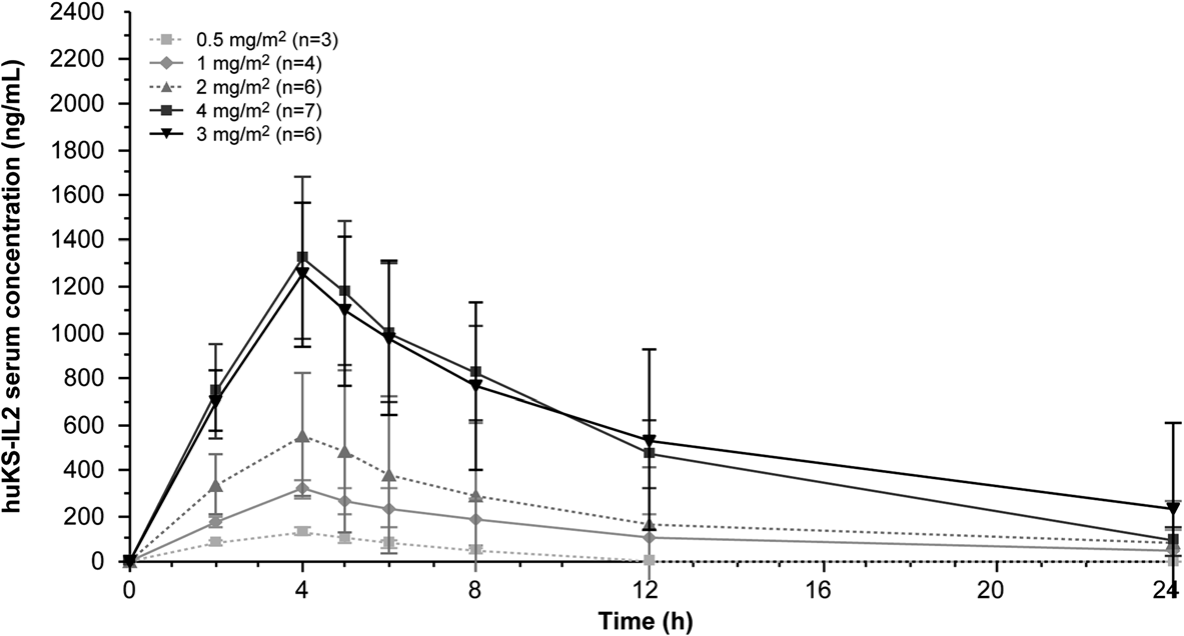
Mean (standard deviation) serum concentration profiles on day 2 of cycle 1.

**Figure 2:**
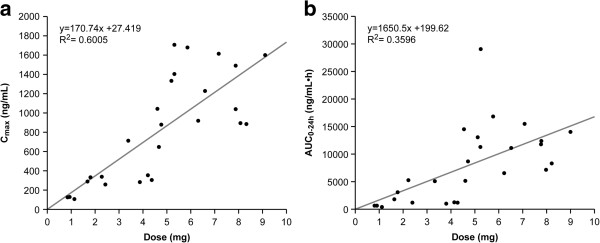
**Dose–response relationship of huKS-IL2. Day 2, cycle 1, as shown by C**_**max**_**(a) and AUC**_**0–24h**_**(b).** C_max_, maximum or peak serum concentration; AUC_0–24h_, area under the concentration versus time curve from time zero to 24 hours after the start of infusion.

Primary huKS-IL2 serum PK parameters on day 2 of cycle 1 after huKS-IL2 first dose administration are summarized in Table 4 by dose cohort. Peak serum concentrations of huKS-IL2 were observed within 1 hour after the end of the 4-hour infusion period (Figure 1). A dose-dependent, but not dose-proportional, increase in C_max_ and AUC_0–24h_ was observed following administration of the first huKS-IL2 doses (Figure 2a and b). There was no clinically relevant drug accumulation observed either within or between cycles. Terminal elimination half-life and systemic clearance did not tend to change with huKS-IL2 dose or over time (data from consecutive cycles on file but not presented here).

**Table 4:** Pharmacokinetic profile of huKS-IL2: serum pharmacokinetic parameters obtained on day 2 of cycle 1

**Parameter**	**huKS-IL2 dosing cohort, mg/m**^**2**^				
	**0.5**	**1.0**	**2.0**	**3.0**	**4.0**
	**(n = 3)**	**(n = 4)**	**(n = 6)**	**(n = 6)**	**(n = 7)**
C_max_, ng/mL					
Mean (%CV)	132.6 (8.9)	316.4 (12.1)	567.6 (52.6)	1252.9 (24.8)	1323.9 (26.9)
Min-Max	119.1–141.0	268.6–350.0	295.4–1052.0	888.1–1712.0	897.3–1687.9
AUC_0-24h_, ng/mL•h					
Mean (%CV)	724.8 (16.5)	2989.7 (59.4)	4850.9 (106.1)	13329.1 (59.7)	12332.5 (28.7)
Min-Max	593.0–826.8	1344.6–5374.7	1184.4–14596.4	6686.3–28917.9	7236.1–16825.5
AUC_0-∞_, ng/mL•h					
Mean (%CV)	823.4 (22.4)	3849.4 (84.9)	6440.9 (128.7)	19769.3 (112.1)	13083.9 (30.0)
Min-Max	627.8–993.9	1406.5–1921.7	1179.8–22682.1	6464.9–64645.7	7418.1–18043.6
t_1/2_, h					
Mean (%CV)	2.7 (20.1)	6.5 (92.4)	4.4 (115.9)	8.5 (100.1)	5.1 (16.4)
Min-Max	2.1–3.2	2.0–13.3	0.9–14.4	3.2–25.6	4.0–6.0
CL, L/h					
Mean (%CV)	1.24 (40.2)	0.84 (75.7)	1.81 (78.4)	0.49 (59.7)	0.66 (41.1)
Min-Max	0.83–1.79	0.26–1.72	0.20–3.27	0.08–0.97	0.32–1.09

Data presented are the arithmetic mean (% coefficient of variation) and minimum–maximum values; *AUC*, area under the concentration–time curve; *AUC*_*0-24h*_, AUC from time zero to 24 hours after the start of infusion; *AUC*_*0-∞*_, AUC from time zero to infinity; *CL*, total body clearance; *C*_*max*_, maximum concentration; *CV*, coefficient of variation; *t*_*1/2*_, apparent terminal half-life.

### Immunogenicity

**Figure 3:**
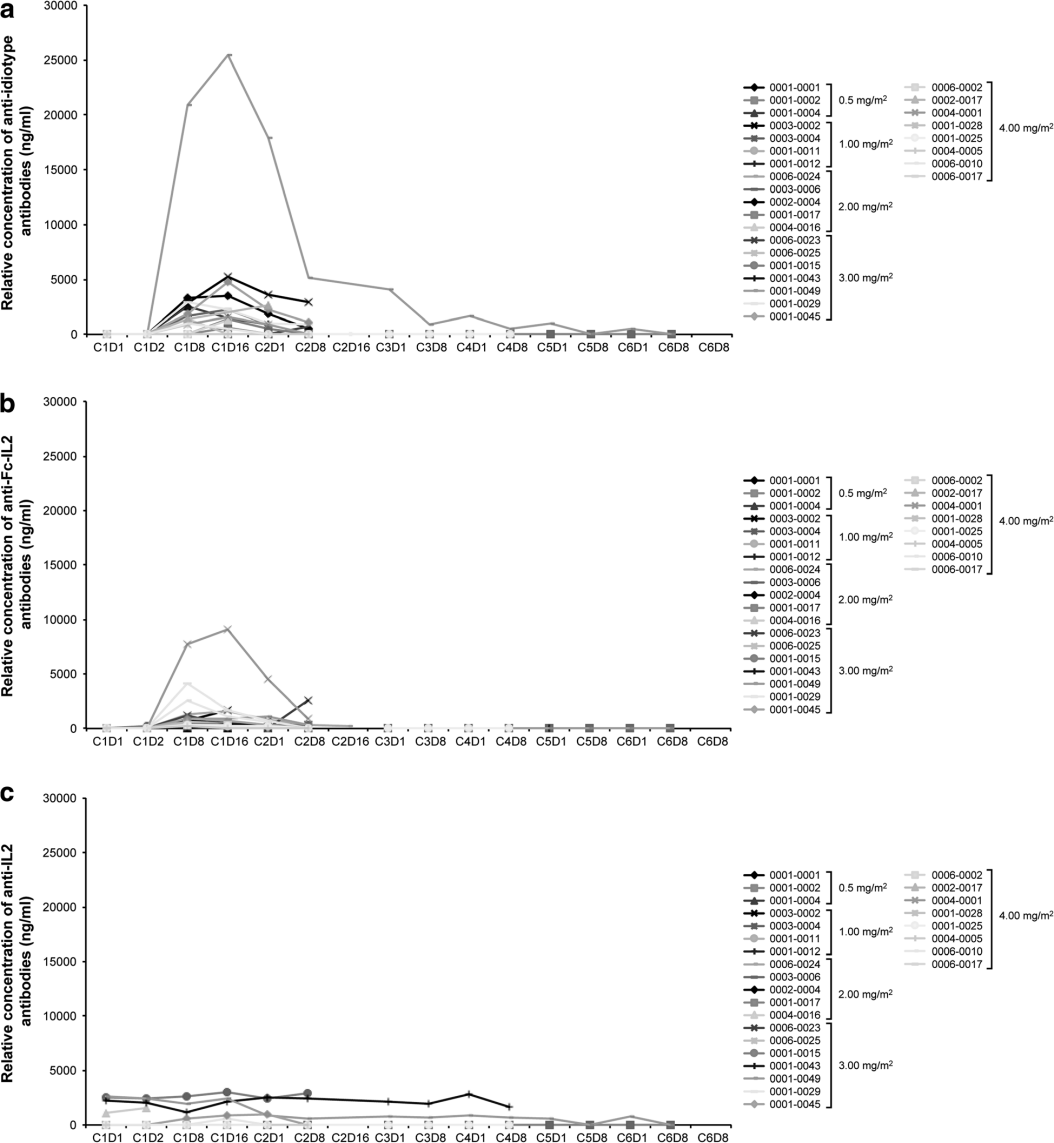
**Serum levels of antibodies over time. **(**a**) Anti-idiotype antibodies; (**b**) anti-Fc-IL2 antibodies; and (**c**) anti-IL2 antibodies.

Immunogenicity samples from 26 patients were available. Overall, 23 of 26 (88.5%) patients treated with huKS-IL2 developed a transient immune response against the drug. In general, and independent of dose cohort, the peak immune response was observed in the first treatment cycle (day 8 or 16) and declined toward baseline values in subsequent cycles (Figure 3). In most patients (n = 14), antibodies against both the complementarity-determining regions (anti-idiotype) and the Fc-IL2 moiety of huKS-IL2 were detected, whereas in 2 patients (1 each in the 1 and 2 mg/m^2^ cohorts) only anti-Fc-IL2 antibodies were found, and 4 patients (2 in the 0.5, 1 in the 3, and 1 in the 4 mg/m^2^ cohorts) exclusively developed anti-idiotype antibodies. In total, only 3 patients (1 in each of the 0.5, 1, and 4 mg/m^2^ cohorts) showed treatment-related antibodies to IL2. In 2 of these 3 patients (1 and 4 mg/m^2^ cohorts), anti-idiotype and anti-Fc-IL2 antibodies were also found, whereas in 1 patient (0.5 mg/m^2^ cohort), the IL2 antibodies were detected concurrently with Fc-IL2 antibodies. The immune response against huKS-IL2 appeared to slightly increase with the dose level, since higher relative concentrations of anti-drug antibodies were observed in the 3.0 and 4.0 mg/m^2^ cohorts.

### Effects on immunologic function

**Figure 4:**
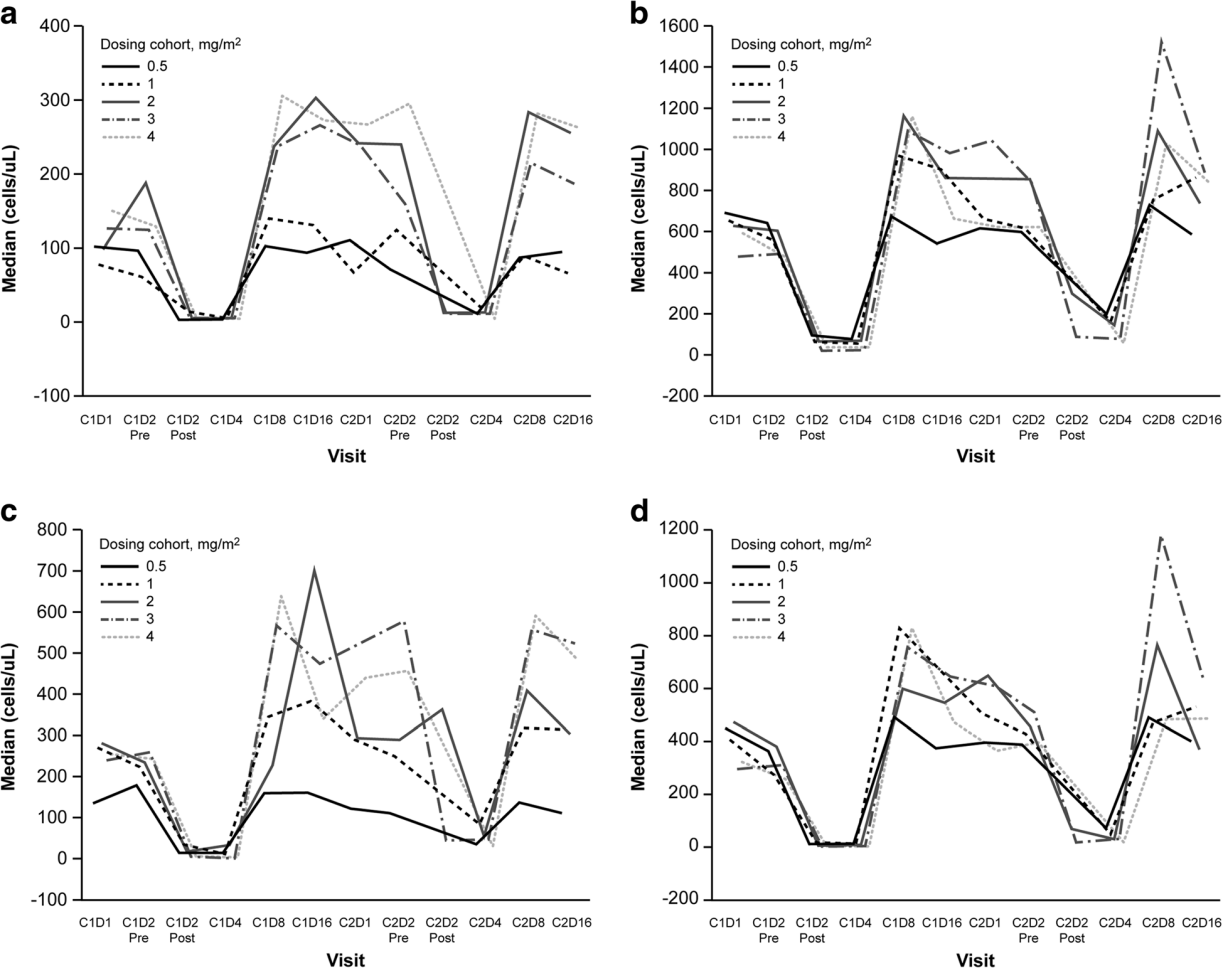
**Immune cell monitoring.** Cytotoxic natural killer cells (**a**); CD4^+^ T-cells (**b**); CD8^+^ T-cells (**c**); and T-reg cells (**d**). C1D1, cycle 1 day 1; C1D2, cycle 2 day 2, etc.

PBMC counts decreased during the first few days of infusion, reflecting treatment-related lymphopenia. For most PBMC populations (cytotoxic NK cells, CD4^+^ T-cells, CD8^+^ T-cells, and T-reg cells), rebound to values higher than baseline was observed at day 8, with return to baseline or near-baseline values by day 1 of the subsequent cycle, again mirroring the temporal pattern of recovery from lymphopenia (Figure 4). Treatment with cyclophosphamide and huKS-IL2 resulted in increased plasma secretory IL2 receptor (sIL2R) expression, with highest values observed at the end of the first week and a return to baseline by the end of the cycle without apparent huKS-IL2 dose-related effects (data not shown). Results of T-cell stimulation with tetanus toxoid did not identify any trends in the magnitude, direction, or pattern of change from day 1 to 8 that was related to the dose of huKS-IL2 (data not shown).

### Clinical response and survival

**Figure 5:**
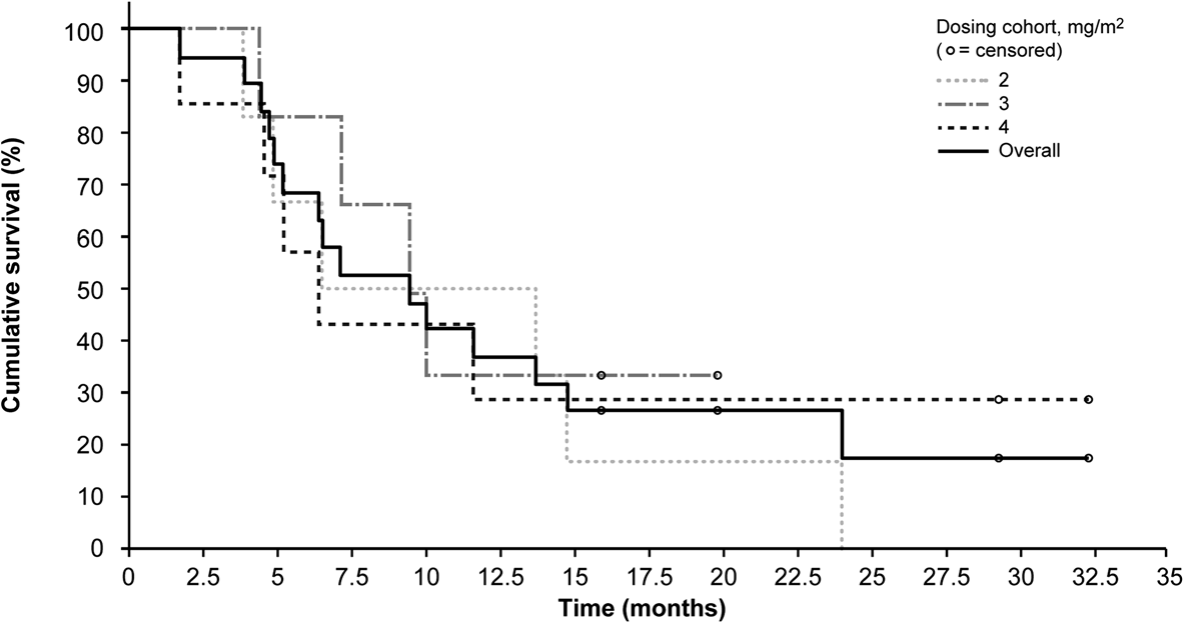
**Kaplan–Meier plot of overall survival.** * The Kaplan–Meier method was applied only for dose-level groups with at least 6 patients.

No objective responses were observed and 10 of 26 (38%) evaluable patients had stable disease as their best response to therapy (Table 5). Stable disease was more frequently observed in the higher huKS-IL2 dosing cohorts (1 of 3 [33.3%] patients in each of the huKS-IL2 0.5 and 1.0 mg/m^2^ cohorts; 2 of 6 [33.3%] in the 2.0 mg/m^2^ cohort, 3 of 6 [50%] in the 3.0 mg/m^2^ cohort, and 3 of 7 [42.9%] patients in the 4.0 mg/m^2^ cohort). In addition, in 3 of 20 patients who received huKS-IL2 at a dose of ≥ 2.0 mg/m^2^, stable disease was observed for 4 or more cycles. Median overall survival was 9.5 months (95% confidence interval, 6.6; 14.8 months) (Figure 5). Seven patients (26%) were alive at the time of this analysis with a median follow-up of 25.8 months (range, 7.4–37.3 months).

**Table 5:** Best overall tumor response

**Best overall response**	**huKS-IL2 dosing cohort, mg/m**^**2**^					
	**0.5**	**1.0**	**2.0**	**3.0**	**4.0**	**Overall**
	**(n=3)**	**(n=4)**	**(n=6)**	**(n=6)**	**(n=7)**	**(n=26)**
	**n (%)**	**n (%)**	**n (%)**	**n (%)**	**n (%)**	**n (%)**
n (missing)	3 (0)	3 (1)	6 (0)	6 (0)	7 (0)	25 (1)
CR	0	0	0	0	0	0
PR	0	0	0	0	0	0
SD	1 (33.3)	1 (33.3)	2 (33.3)	3 (50.0)	3 (42.9)	10 (40.0)
PD	2 (66.7)	2 (66.7)	4 (66.7)	2 (33.3)	4 (57.1)	14 (56.0)

Notes: Includes subjects who withdrew due to PD prior to an objective tumor response assessment. One subject discontinued the study due to treatment-emergent adverse events without any post-baseline disease assessment.

*CR*, complete response; *PD*, progressive disease; *PR*, partial response; *SD*, stable disease.

## Discussion

With dose escalation up to 4 mg/m^2^/day × 3 days per cycle, we identified an MTD of 3.0 mg/m^2^/day. This dose is substantially lower than the MTD of 6.4 mg/m^2^/day determined in a previous phase 1 trial of huKS-IL2 as single-agent therapy in patients with prostate cancer [19]. In that study, 4 of 22 patients experienced DLTs (dyspnea, hypotension, symptoms related to a vascular-leak syndrome: hypotension, chills, rigors, and crackling at the base of the lung; hypertension, anemia, and asthenia) [19]. In the current study, DLTs were observed in 4 of 27 patients. At the highest dose level of 4.0 mg/m^2^, dose-limiting myleosuppression occurred in 2 patients. Hypoxia, dyspnea, bronchospasm, and GGT increase were also observed. However, we could not identify a consistent pattern of toxicity, and typical signs of a systemic vascular-leak syndrome were not detected. Profound (≥ grade 3) non-dose-limiting lymphocytopenia was observed in 5 patients (at a dose ≥ 2.0 mg/m^2^ in 4 patients). Consistent with other IL2/cytokine-based therapies, these episodes of lymphopenia may not reflect myelosuppression, but are likely due to immunocytokine-induced lymphocyte trafficking to the peripheral tissues [20], including tumor beds or draining nodal tissues.

The estimated huKS-IL2 C_max_ and AUC_0–24h_ values varied widely within and between huKS-IL2 dosing cohorts, within cohort coefficients of variation ranging from 8.9% to 52.6% for C_max_ and from 16.5% to 106.1% for AUC_0–24h_. A dose-dependent, but not dose-proportional, increase in C_max_ and AUC_0–24h_ was observed for huKS-IL2 over the tested dose range. C_max_ was achieved within 1 hour after the end of the 4-hour infusion period. No clinically relevant accumulation of huKS-IL2 was observed either within (ratio for AUC_0–24h_ ranging from 0.69 to 1.37) or between (ratio for AUC_0–24h_ ranging from 0.55 to 1.23) treatment cycles. Terminal half-life and systemic clearance did not tend to change with dose or over time. These PK results are consistent with those obtained in previous clinical studies [19], in which C_max_ was also achieved within 1 hour after infusion end and displayed a dose-dependent, but not dose-proportional, increase in concentrations and exposure.

There appear to be some similarities in the PK activity of immunocytokines. Similarities between huKS-IL2 and published PK data for hu14.18-IL2 (EMD 273063), a humanized anti-GD2 monoclonal antibody linked to IL2, included that C_max_ was achieved at the end of the 4-hour infusion period, increases in C_max_ and AUC were dose dependent, and there was no accumulation of the immunocytokines [21]. However, in contrast to huKS-IL2, a dose-dependent decrease in clearance was observed with hu14.18-IL2, and both clearance and mean peak concentrations increased in cycle 2 [22]. These differences may be related to the specific target antigens.

Most patients treated with huKS-IL2 had a transient immune response, commonly anti-idiotype or anti-linker (the engineered component that links the IL2 to the antibody) antibody induction. Whether or not these antibody responses have any detrimental/neutralizing effects on huKS-IL2 is not known. As in other antibody-based therapies, it is also possible that the induction of anti-idiotype antibodies could enhance the anti-EpCAM effects via downstream antibody responses. In a monotherapy phase 1 trial, immunologic activity of huKS-IL2 was demonstrated by increases in lymphocyte counts, NK cell activity, and antibody-dependent cellular cytotoxicity [19]. In the current study, we monitored immune response to therapy by serial flow cytometric evaluation of PBMC populations (T-cell subsets, NK cells, and T-reg cells), serum-soluble IL2 receptor determination, and PBMC response to tetanus toxoid. Consistent with the effects on immune function of exogenous IL2, PBMC counts decreased during the days of huKS-IL2 infusion, reflecting treatment-related lymphopenia/lymphocyte trafficking. Rebound of PBMC populations to baseline levels, and often higher, was observed within a week of the end of infusion, with rebound appearing to be more pronounced at higher huKS-IL2 doses. In contrast, there was no evidence of dose-dependent huKS-IL2 effects on the increase in sIL2R levels observed *in vivo* or the PBMC response to tetanus toxoid *in vitro*.

The rationale for combining huKS-IL2 with low-dose cyclophosphamide in the present study was based on the earlier observation that certain cytotoxic chemotherapeutic agents sensitize tumors to the tumoricidal effects of the immunocytokine within the tumor microenvironment [18]. Sensitization may occur as a result of reducing intra-tumoral pressure and lowering the macromolecular diffusion barrier, thus potentially permitting increased uptake of the immunocytokine antibodies. Moreover, existing clinical evidence suggests the combination of low-dose cyclophosphamide together with a continuous-infusion or IL2 or other related biologics yields significant anti-tumor activity in patients with advanced cancers [23,24]. These effects have been shown to be related to differential loss of regulatory T-cells compared with cytotoxic lymphocyte populations.

In a study that evaluated the cancer vaccine IMA901 in patients with renal cell carcinoma, pre-treatment with a single dose of cyclophosphamide was shown to down-modulate T-regs and contribute to overall survival [25]. Patients receiving cyclophosphamide had a significant decrease of median T-reg levels 3 days after cyclophosphamide treatment. In the current study we cannot specifically comment on the status of T-reg cells in response to the cyclophosphamide alone, as the addition of huKS-IL2 on days 2–4 resulted in loss of T-reg cells followed by rebound, as would be expected with IL2 effect. However, a previous study that evaluated recombinant IL2 (rIL2) and low-dose cyclophosphamide in patients with malignant melanoma and renal cell carcinoma reported that the regimen had minimal anti-tumor activity—no remission in renal cell carcinoma patients and partial or minor response in 3 (17%) melanoma patients [26]. An additional 4 patients had stable disease (1 with renal cell carcinoma and 3 with melanoma).

Based on pre-clinical *ex vivo* studies and animal models, it is likely that immunocytokines have clinical effects by both NK cell- and T-cell-mediated mechanisms [27,28]. The immunocytokines facilitate antibody-dependent cellular cytotoxicity (ADCC) *in vitro* with both healthy donor effector cells and with immune cells from patients with advanced cancer. Similarly, depletion of NK cells dampens the anti-tumor activity of immunocytokines in mouse models. Although NK cells play a role in immunocytokine therapy, pre-clinical data also support the key role of T-cell-mediated effects, including the development of memory that is protective against subsequent tumor challenge. Therefore, it is theorized that immunocytokine therapeutic effects result from NK-mediated ADCC of tumor cells that contributes to direct tumor cell death, and that this effect results in release of antigens that can facilitate/potentiate the development of anti-tumor T-cell populations. These T-cells can then act as primary anti-tumor effectors as well as providing long term memory effects. The effects on T-reg cells and the potential benefit in terms of induction of T-cell immunity was one of the main rationales for the use of cyclophosphamide in this study.

With regard to anti-tumor activity in the current study, no objective responses were observed. Ten patients had stable disease as best response and 7 patients were alive at the time of analysis. As this was a phase 1 study, the primary objective was not to evaluate response to therapy. The clinical utility of immunotherapy in general has been best seen in the setting of minimal residual or sub-clinical disease. For this reason it was not expected that huKS-IL2 therapy (alone or with low-dose cyclophosphamide) would demonstrate clinical responses in patients with end stage clinically measurable (and often bulky) disease. Although not studied as a specific endpoint, several subjects have had excellent responses to additional cytotoxic therapies and have become long-term survivors. Granted, the number of cases is very small; however, no pattern or distinctive features such as pre-treatment lymphocyte patterns are seen in these subjects. At this time continued follow-up on this handful of cases is of particular interest.

For these reasons, one could argue that both the higher-dose monotherapy and the combination of cyclophosphamide with lower doses of huKS-IL2 should/could be developed further. A potential next step that could address these issues would be to move to a randomized phase 2 study of both regimens, with anti-tumor effect as the primary outcome. Clearly a study such as this would require close monitoring and early stopping strategies in case one regime is found to be more effective.

## Conclusions

In this trial of low-dose cyclophosphamide and huKS-IL2 in patients with advanced solid tumors, the combination was shown to be feasible and safe. MTD was determined to be 3 mg/m^2^/day. A substantial proportion of patients across dose levels experienced stable disease, with 3 patients (at dose level ≥2.0 mg/m^2^) experiencing stable disease for ≥4 cycles. These findings may warrant further investigation of the anti-tumor activity of huKS-IL2 in combination with low-dose cyclophosphamide as a potential immunotherapy for patients with EpCAM-positive solid tumors.

## Abbreviations

ADCC: Antibody-dependent cellular cytotoxicity; AEs: Adverse events; AUC: Area under the concentration–time curve; AUC0–∞: AUC from time zero to infinity; AUC 0–24h: AUC from time zero to 24 hours after the start of infusion; CL: Total body clearance; Cmax: Maximum concentration; CR: Complete response; CV: Coefficient of variation; DLT: Dose-limiting toxicity; ELISA: Enzyme-linked immunosorbent assay; EpCAM: Epithelial cell adhesion molecule; GGT: Gamma-glutamyltransferase; huKS-IL2: Humanized KS-interleukin-2; IL2: Interleukin-2; IRB: Institutional Review Board; MTD: Maximum tolerated dose; NCI CTCAE v3.0: National Cancer Institute Common Terminology Criteria for Adverse Events version 3.0; NK: Natural killer; PBMC: Peripheral blood mononuclear cell; PD: Progressive disease; PK: Pharmacokinetic; PR: Partial response; RECIST: Response Evaluation Criteria in Solid Tumors, version 1.0; rIL2: Recombinant interleukin-2; SD: Stable disease; sIL2R: Secretory IL2 receptor; t1/2: Terminal half-life; TEAE: Treatment-emergent adverse event; ULN: Upper limit of normal; WBC: White blood cell

## Competing interests

Joseph Connor is a consultant for EMD Serono; Nancy L. Lewis is a consultant for Macrogenics Inc.; Philip B. Komarnitsky was an employee of EMD Serono Inc. at the time the study was conducted; Maria R. Mattiacci was an employee of Merck Serono at the time the study was conducted; Jean Henslee-Downey is an employee of EMD Serono Inc.; Daniel Kramer and Roland Neugebauer are employees of Merck KGaA; Mihaela C. Cristea, Lionel D. Lewis, Mildred Felder, Sarah Stewart, Josephine Harter, and Roger Stupp have nothing to disclose.

## Authors’ contributions

JPC, PBK, JH-D, MRM, DK, and RN conceived and designed the study. JPC, MCC, NLL, LDL, MF, SS, JH and RS acquired the data. MRM performed the statistical analysis of the safety and efficacy results; RN performed the statistical analysis of the PK data; DK performed the statistical analysis of the immunogenicity data. All authors analyzed and interpreted the data, contributed to manuscript drafting, critically reviewed the manuscript, and approved the final manuscript.

## Pre-publication history

The pre-publication history for this paper can be accessed here:

http://www.biomedcentral.com/1471-2407/13/20/prepub
